# Adapting Pediatric Anesthesia Education to Generation Z Learners: A Narrative Review

**DOI:** 10.7759/cureus.104964

**Published:** 2026-03-10

**Authors:** Barkha D Agrawal, Revanth B. Challa, Nand Kishore Joshi, Mangesh Mulaokar, Ashutosh Kumar

**Affiliations:** 1 Anaesthesiology, All India Institute of Medical Sciences, Nagpur, Nagpur, IND

**Keywords:** education, medical, pediatric anesthesia, professional competence, simulation training

## Abstract

Pediatric anesthesiology is a subspecialty with high complexity requiring advanced technical skills, rapid decision-making, and empathetic communication. Children present with distinct physiological and anatomical vulnerabilities, and several studies suggest that traditional training models largely based on observation and supervised participation may not consistently provide sufficient hands-on exposure, particularly for rare but critical events. Concurrently, a generational shift has reshaped medical education, with Generation Z (Gen Z) trainees demonstrating preferences for interactive, technology-supported, and feedback-rich learning environments.

This narrative review, based on a focused synthesis of literature from PubMed, Cochrane Library, and Google Scholar (2000-2025), integrates evidence from both pediatric anesthesia-specific studies and broader medical education literature to examine how contemporary educational strategies align with evolving learner characteristics. Findings suggest that the most effective approach is not reliance on any single innovation but a thoughtful integration of experiential learning, structured simulation, digital reinforcement, and mentorship within competency-based frameworks. When implemented alongside supervised clinical exposure, these approaches may enhance skill acquisition, engagement, and preparedness.

Aligning pediatric anesthesia education with modern learning science and generational needs is, therefore, timely and necessary. Carefully designed learner-centered training models may strengthen competency development while upholding ethical principles such as “training without harm,” ultimately preparing anesthesiologists to deliver safe, compassionate, and high-quality care to children.

## Introduction and background

Pediatric anesthesiology involves perioperative care for infants, children, and adolescents whose physiology differs markedly from that of adults, placing them at higher anesthetic risk. Anatomical features such as a larger occiput, cephalad larynx, and floppy epiglottis make airway management more challenging, while higher oxygen demands further increase vulnerability. Consequently, respiratory complications account for about 43% of pediatric adverse events and nearly half of anesthesia-related pediatric deaths, exceeding adult rates [1-3]. Pediatric patients also experience pharmacological variations in terms of drug metabolism due to immature organs. Additionally, pediatric patients often require individualized psychosocial intervention to reduce anxiety and increase patient trust. All these unique needs of neonates, infants, and children require skills and knowledge beyond those learned in an anesthesiology residency.

Most anesthesia residency programs have a pediatric anesthesia rotation as part of their training program. The pediatric anesthesia rotation provides trainees with knowledge related to airway management, resuscitation, pharmacology, and child psychology. A recent cross-sectional survey of anesthesia trainees indicated that more than half of respondents reported assisting with pediatric cases; however, fewer than 20% of trainees reported having cared for patients younger than one year of age [4]. Limited exposure to infants and high-risk pediatric cases may affect trainee readiness for independent practice. Limited hands-on experience with physiologically vulnerable patients can reduce confidence in airway management, crisis recognition, and real-time decision-making. This gap highlights the need for structured simulation and competency-based training to supplement clinical exposure and strengthen preparedness in pediatric anesthesia.

A broader generational transition is occurring across educational settings, with Generation Z (Gen Z) representing a growing proportion of learners, reflecting trends described in both sociological and health professions education literature. Gen Z (1995-2012), comprising most medical school students and junior anesthesia residents, is the first generation to grow up entirely in a digital environment. As a result, the learning style preferences of Gen Z are distinctly different from traditional lecture-based and hierarchical learning models. Digital natives' preferred learning modalities include interactive content, short-form content, visual content, and access via mobile devices. Furthermore, Gen Z responds positively to educational technologies that allow for experiential and relational components, such as early clinical exposure, team-based learning, and mentorship, as these promote psychological safety, relationship building, and a sense of purpose.

This review is different from other educational reviews that look at generational learning trends in general medical settings. Instead, it looks at how these trends affect pediatric anesthesiology, which is a subspecialty with its own set of physiological challenges, high-risk airway management, and ethical issues. This review provides specialty-focused assistance by directly connecting contemporary educational practices to pediatric-specific clinical difficulties, rather than offering general pedagogical recommendations. Its purpose is to enable teachers to carefully change pediatric anesthesia training programs to meet the needs of today's students while still keeping patient safety and clinical rigor. By developing educational strategies that are aligned with the learning styles, interests, and life experiences of Gen Z trainees, we hope to enable them to become competent professionals who deliver safe and compassionate anesthesia care to the children and families they will serve.

## Review

Methodology

This manuscript was conducted as a narrative educational review employing an interpretive synthesis approach. Unlike systematic or scoping reviews designed to exhaustively aggregate or quantitatively appraise evidence, this review seeks to conceptually integrate literature on generational learning theory with contemporary pediatric anesthesia education practices.

A focused literature search was performed in PubMed, the Cochrane Library, and Google Scholar for publications between January 2000 and October 2025. Search terms included combinations of “pediatric anesthesia,” “education,” “Generation Z,” “simulation,” “microlearning,” “flipped classroom,” “gamification,” “adaptive learning,” “mentorship,” and “interprofessional education.” Reference lists of relevant articles were manually screened to identify additional sources.

Titles and abstracts were screened for relevance to pediatric anesthesia training, generational learning characteristics, or educational innovation. Articles were included if they addressed educational strategies applicable to pediatric anesthesia or provided conceptual insight into contemporary learner characteristics. Eligible publications comprised empirical studies, systematic and narrative reviews, consensus statements, and educational frameworks. Commentaries lacking educational focus, non-English publications, and studies unrelated to training methodologies were excluded. Full texts were reviewed for articles meeting preliminary relevance criteria. Selection decisions were guided by conceptual contribution and thematic alignment rather than methodological hierarchy, consistent with narrative review methodology.

The review process involved structured identification, title and abstract screening, full-text eligibility assessment, and thematic inclusion of relevant studies, following Preferred Reporting Items for Systematic Reviews and Meta-Analyses (PRISMA)-style reporting principles adapted to the interpretive framework of a narrative synthesis.

Given the limited volume of pediatric anesthesia-specific educational research, relevant evidence from broader health professions education literature was included to provide contextual and conceptual support. Thematic synthesis was guided by generational learning theory, adult learning principles, competency-based education, and experiential learning frameworks to examine how contemporary instructional strategies align with evolving trainee characteristics.

Components of planned systematic development of pediatric anesthesia education for Gen Z pediatric anesthesia trainees

**Figure 1:**
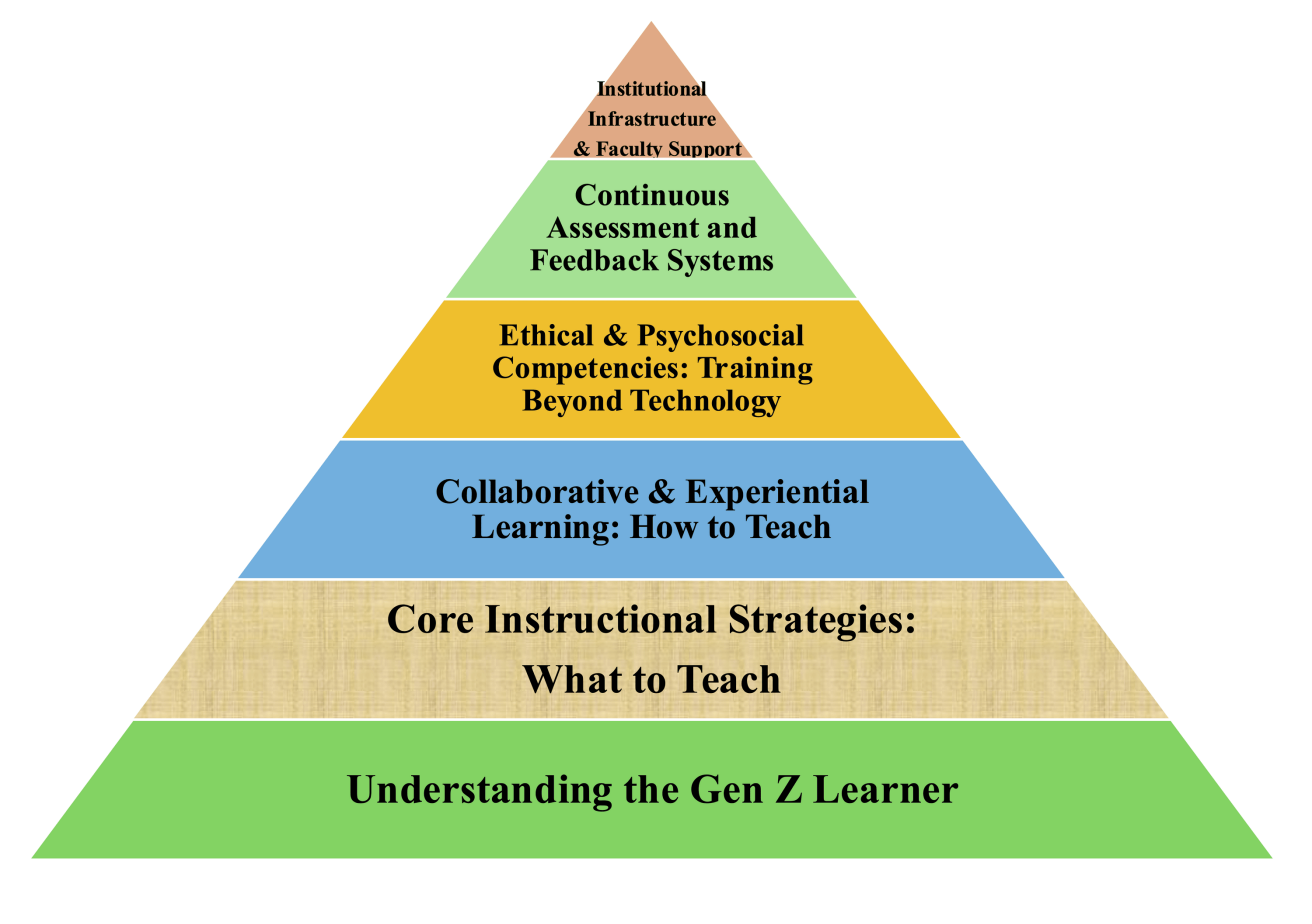
Components of Planned Systematic Development of Pediatric Anesthesia Education Image credits: Created by the authors using Microsoft PowerPoint (Microsoft Corp., Redmond, WA, USA). Gen Z: Generation Z

Teaching methods for Gen Z pediatric anesthesia trainees should reflect their psychological traits and be thoughtfully planned. The next six sections outline a practical, learner-centered framework for developing Gen Z pediatric anesthesia trainees utilizing state-of-the-art pedagogical strategies, technology-enhanced learning, and development of morality and institutional structure (Figure 1). Together, these components provide a systematic approach to develop experiential learning opportunities that meet the needs of Gen Z pediatric anesthesia trainees to participate in and succeed in learning experiences that will prepare them to become proficient pediatric anesthetists.

Learning Characteristics of Gen Z

Current anesthesiology residents comprise mainly the Gen Z population. Gen Z is technologically adept, prefers face-to-face communication, and is highly visual. Over 90% of Gen Z students report that they are "heavy users of technology" and "digital natives." Gen Z trainees prefer to learn through multimedia, rather than traditional lectures or textbooks. They tend to lose interest during long lectures; however, short video clips, podcasts, and mobile applications allow them to learn at their own pace and in their own time. Approximately 91% report that technology directly impacts how they learn, and they prefer short, visually rich formats to rapidly assimilate information [5-7].

Gen Z trainees value efficiency and the ability to access information anytime, anywhere. Gen Z trainees can utilize e-learning platforms and interactive modules to learn while at work or en route. Gen Z trainees show interest in the opportunity to collaborate and receive rapid feedback, as they were raised in a continually connected world. They thrive in interactive, peer-led settings that resemble social media and can expect immediate feedback. Frequent, formative debriefings after simulations or rapid bedside feedback are much more effective in motivating trainees than evaluations provided post event. Gen Z trainees also learn best through seeing and performing and therefore prefer short instructional videos, animations, or hands-on scenarios that show the relevance of their learning in actual care.

In summary, Gen Z pediatric anesthesia trainees function best in learning environments that are flexible, provide continuous feedback, and incorporate technology. The comparison of generational learning characteristics of Gen Z and Millennials and their implications in pediatric anesthesia is enumerated in Table 1. The generational characteristics described should be interpreted as broadly discussed trends rather than universally validated traits and are presented here as a conceptual framework to contextualize evolving learner expectations.

**Table 1:** Generational Learning Characteristics and Implications for Pediatric Anesthesia Education This table is a conceptual synthesis developed by the authors to summarize generational learning characteristics and their potential implications for pediatric anesthesia education. The table content was derived from themes discussed in the cited educational and generational learning literature referenced throughout the manuscript. The authors used ChatGPT, version 5.2 (OpenAI, San Francisco, CA, USA) for assistance with language formatting only.

Aspect	Generation Z (1995–2010)	Millennials (1981–1996)	Implications for Pediatric Anesthesia Education
Technology Use and Flexibility	Grew up in fully digital environments; accustomed to mobile, on-demand, multimedia content; expect rapid information access	Transitioned from analog to digital systems; comfortable with blended formats but accustomed to structured schedules	Use blended, mobile-friendly platforms with flexible self-paced modules and on-demand pediatric anesthesia resources
Learning Style and Attention Span	Prefer concise, visually rich, and interactive content; may demonstrate reduced tolerance for prolonged passive lectures	More accustomed to traditional didactic sessions with clearly defined objectives	Use flipped classrooms, case-based discussions, and structured micro-modules to reinforce pediatric concepts efficiently
Collaboration and Social Learning	Value peer interaction, rapid feedback, and socially connected learning environments	Appreciate teamwork but often prefer instructor-guided discussions	Incorporate team-based learning (TBL), peer simulations, and group discussions with real-time debriefing
Motivational Drivers	Engage strongly with content that demonstrates clinical relevance, measurable progress, and real-time feedback	Often motivated by mentorship, professional advancement, and long-term career goals	Explicitly link training to pediatric patient outcomes, safety benchmarks, and competency milestones
Mentorship Expectation	Prefer accessible, coaching-style mentorship with frequent formative feedback	Expect periodic structured evaluation and traditional mentor–mentee hierarchies	Establish structured mentorship programs with regular formative feedback and reflective debriefings
Experiential Learning and Simulation	Favor early hands-on engagement and simulation before independent responsibility	Typically accept graduated autonomy beginning with observation	Provide a staged simulation-based rehearsal followed by supervised pediatric clinical exposure

Modern teaching methods for pediatric anesthesia training

**Figure 2:**
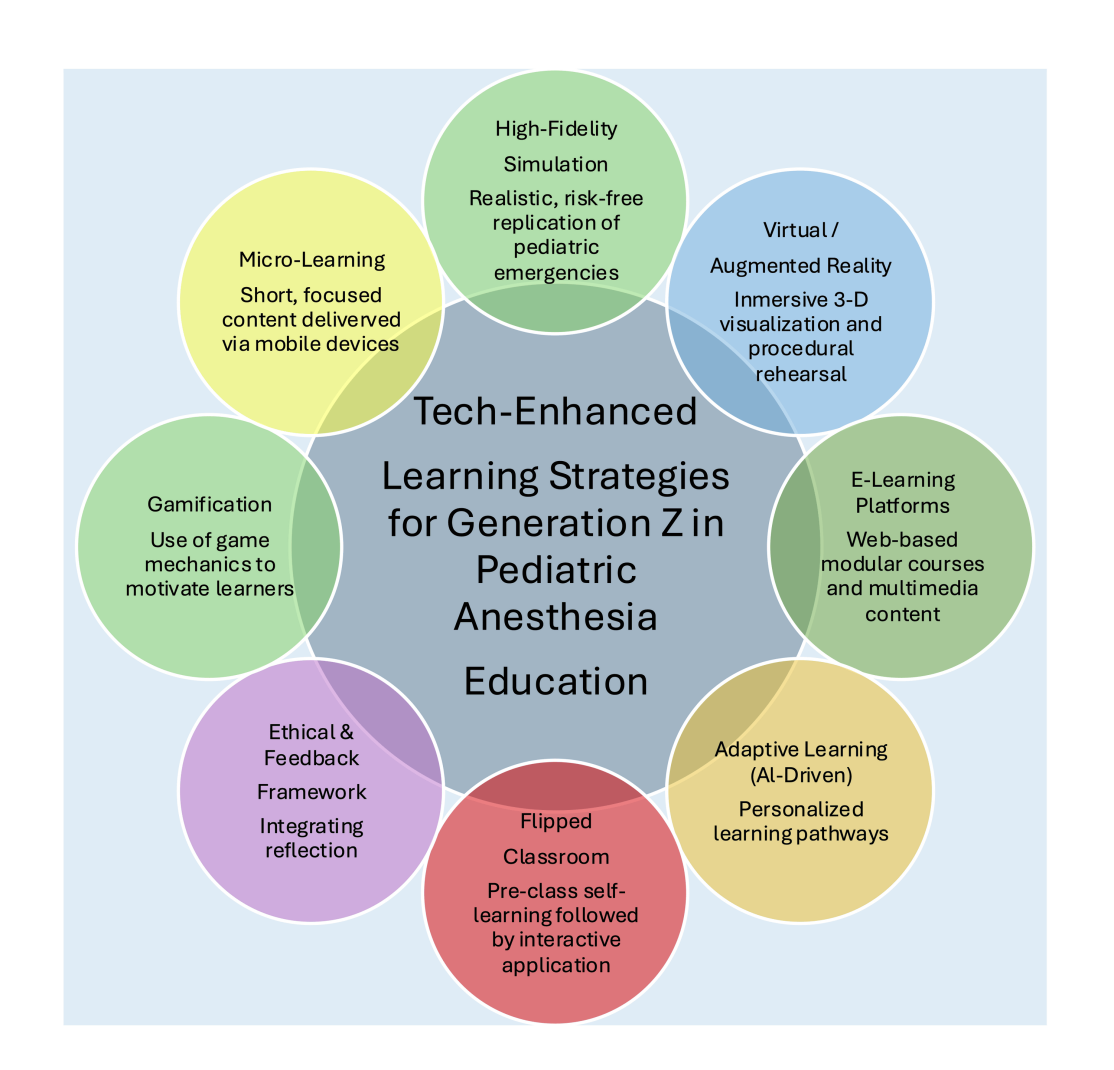
Tech-Enhanced Learning Strategies for Generation Z Image credits: Created by the authors using Microsoft PowerPoint (Microsoft Corp., Redmond, WA, USA).

Modern pediatric anesthesia education should adapt to accommodate Gen Z’s preferred styles of learning, thus implementing a learner-centered approach to teaching. Important modern educational modalities for pediatric anesthesia training include high-fidelity simulation, virtual reality/augmented reality (VR/AR), e-learning modules, artificial intelligence (AI)-driven adaptive learning systems, flipped classrooms, microlearning, and gamification (Figure 2). When implemented individually and collectively, these methods facilitate the creation of a more interactive, individualized, and accessible learning environment for pediatric anesthesia trainees. The following section reviews the effectiveness of each modality to instruct pediatric anesthesia trainees.

Simulation-Based Education

Simulation is an integral part of contemporary anesthesia education, particularly in pediatric patients, as there are few critical incidents in pediatric anesthesia, but they require expert management. Repetitive simulated exposures allow trainees to acquire technical and crisis management skills, such as leadership, teamwork, and communication. Importantly, they can learn from trial and error without harming children.

There are various types of simulation. For example, in situ simulations improve teamwork and system awareness, while VR modules improve the ease and enjoyment of tasks such as fiber-optic bronchoscopy and epidural placement [8]. Standardized patient simulations improve communication skills, particularly in obtaining informed consent from parents. The ethical principle of “no harm” implies that psychological safety and appropriate debriefing processes must be implemented, especially in emotionally charged situations such as pediatric cardiac arrest. Simulation provides a safe, realistic, and engaging educational experience, particularly advantageous for Gen Z trainees who excel in interactive, technology-enhanced learning environments. Furthermore, simulation transitions the traditional “see one, do one, teach one” model to a more structured model of “learn, simulate, and perform under supervision” [9]. Strong simulation programs have been demonstrated to reduce complications and ICU admissions, leading to improved outcomes for patients [10]. Recent literature identifies simulation-based education as a foundational and widely adopted element of pediatric anesthesia training [11]. A “boot camp” orientation program using simulation and other active-learning techniques for incoming pediatric anesthesia fellows rapidly builds clinical skills, teamwork, and confidence [12]. However, the high financial cost, infrastructure requirements, and variability in debriefing quality may limit equitable access and consistent effectiveness across institutions. Furthermore, while simulation improves confidence and short-term performance metrics, direct evidence linking simulation exposure to long-term pediatric patient outcomes remains limited.

Implications for pediatric anesthesia: High-fidelity simulation employs advanced mannequins or computer-generated models that mimic pediatric physiology, allowing safe practice of emergency scenarios such as infant airway obstruction, neonatal cardiac arrest, anaphylaxis, or hemodynamic collapse in congenital heart disease [4,13]. The Society for Pediatric Anesthesia and other organizations continue to create courses and scenarios specific to pediatric anesthesia [14]. Simulation bridges the gap in experience, especially in low-volume centers, by creating low-frequency, high-risk scenarios such as cardiac arrest due to laryngospasm or situations where one cannot intubate or ventilate [4]. Simulation has proven to enhance the skills of pediatric anesthesia residents in high-crisis situations, leading to better preparedness in managing critical life situations [12].

E-learning and Flexible Digital Platforms

E-learning has become an important component of modern medical education, including pediatric anesthesia training. Multimedia modules, interactive cases, and digital resources allow trainees to access content flexibly and reinforce core concepts at their own pace. Evidence from broader health professions education suggests that well-designed e-learning can improve knowledge acquisition and learner satisfaction compared with traditional lectures, particularly when interactive elements are included. However, much of this evidence derives from general medical education rather than pediatric anesthesia-specific studies, and long-term clinical outcome data remain limited.

Adaptive e-learning platforms further personalize instruction by identifying performance gaps and delivering targeted content. While promising, these systems require careful validation and integration within structured curricula. Importantly, digital tools should complement, not replace, supervised clinical exposure and mentorship, as excessive reliance may contribute to digital fatigue and reduced interpersonal engagement.

Implications for pediatric anesthesia: A pediatric anesthesia module may reveal areas where the learner needs more support, such as in neonatal opioid dosing, and provide targeted educational materials or assessments until the trainee demonstrates competence. The platform evolves with the learner’s growing skillset, keeping track of their progress across various domains (such as identifying weaknesses in regional anesthesia, drug dosing, etc.) and helping learners design study plans that fit their needs [7]. A Delphi consensus study gathered expert input to update pediatric anesthesia training [15]. This serves as a roadmap for aligning pediatric anesthesiology education with contemporary needs and Gen Z learning styles [16]. In addition, the PediCrisis app and other clinical tools contribute to this ecosystem by offering algorithm-based checklists for emergency scenarios such as laryngospasm, anaphylaxis, or cardiac arrest. Demonstration videos of clinical exams and procedures were among the most frequently accessed materials. Ultimately, these tools help trainees feel more ready to handle emergencies and act as a bridge between digital learning and real-world clinical practice.

Flipped Classroom

The flipped classroom model frees up classroom time by shifting foundational content to pre-class preparation, enabling more interactive practice during sessions. The subsequent sessions can typically be comprised of case-based discussions, skill demonstrations, and crisis management drills. This setup works particularly well for Gen Z, who enjoy visual media, doing things actively, and learning alongside peers. Studies in medical education have consistently demonstrated that flipped classroom courses enhance retention, critical thinking, and learner engagement [17]. A comprehensive meta-analysis of 141 studies comparing flipped classrooms to traditional lectures in medical education. It found that flipping the classroom yields significantly better knowledge outcomes and higher student satisfaction [18]. Nevertheless, the flipped model depends mainly on learner self-discipline and adequate preparation; without structured accountability, knowledge gaps may persist. Evidence supporting sustained long-term retention beyond immediate engagement outcomes also remains variable.

Implications for pediatric anesthesia: In pediatric anesthesia, pre-recorded lectures, animations, or interactive e-modules provide trainees with brief didactic presentations related to managing a child’s airway, administering fluids, and using a variety of medications in pediatric anesthesia. These modules encourage trainees to learn with and from each other and prepare for high-stakes clinical pediatric anesthesia environments.

Microlearning and Modular Learning

Microlearning includes breaking down learning into short, focused lesson segments created to meet a clear purpose. Microlearning works very well for Gen Z trainees who prefer short, high-impact digital content, multitasking, and learning on the go. It succeeds due to its reliance on spaced repetition and reinforcement. Owing to trainees’ active lifestyles, microlearning modules must be brief and schedule-friendly. Educators can thoughtfully design microlearning modules into an integrated curriculum to prevent fragmentation. Microlearning’s focus on speed and progress correlates with Gen Z’s motivation to remain engaged. A non-randomized clinical trial conducted in 2025 evaluated microlearning via Instagram™ “Reels” (90-second videos) for third-year medical students learning neuroanatomy. In this study, the authors concluded that bite-sized social media videos can enhance short-term knowledge acquisition and engagement, but sustained long-term benefits likely require additional reinforcement strategies. This implies that Gen Z learners enjoy and learn from micro-content (aligning with their shorter attention spans and mobile learning habits), but we should be mindful that such tools should complement, but not replace, deeper learning experiences [19]. But its effectiveness as a standalone strategy for higher-order clinical reasoning remains uncertain.

Implications for pediatric anesthesia: Microlearning modules can provide pediatric anesthesia trainees with the fundamentals of pediatric anesthesia with minimal effort. Programs can develop three-to-five-minute modules similar to “Recognizing and Managing Malignant Hyperthermia in Children,” “Determining Pediatric Endotracheal Tube Sizes,” and “Checking Pediatric Anesthesia Machines.” After initial development, modules can be distributed as interactive slides or videos sent to trainees weekly. Over time, micro modules can provide a wealth of knowledge regarding pediatric anesthesia. Micro-modules can also assist in reinforcing the acquisition of pediatric anesthesia concepts through the use of short quizzes, flashcards, or infographics, such as a color-coded Pediatric Advanced Life Support (PALS) algorithm. Organizing micro-modules into thematic categories (e.g., pediatric airway management, perioperative analgesia) assists in enhancing comprehension of underlying principles.

Gamification and Interactive Learning

Gamification provides a variety of practical benefits to pediatric anesthesia educators, particularly with regard to promoting active participation in high-risk, low-frequency pediatric anesthesia-related scenarios that cannot be encountered clinically. Simulator games can become “game missions” to increase engagement. Each success earns clues to “saving” the child, combining clinical realism with a playful framework that boosts stress resilience and teamwork [20]. A systematic review of 23 studies on gamification and game-based learning in medical education concluded that gamification in medical education often increases residents' enthusiasm and participation, but future designs should strive to foster higher-order thinking and not just rote learning. In short, game elements (e.g., quizzes, leaderboards, and scenario challenges) appeal to Gen Z’s competitive and social nature, but to be truly effective, such strategies must be carefully aligned with educational objectives to produce deep learning [21]. Additionally, poorly aligned gamified interventions may inadvertently highlight competition over mastery, and existing research connecting gamification to sustained clinical competence or enhanced patient outcomes is inadequate.

Implications in pediatric anesthesia: Gamification also allows trainees to develop rapid decision-making skills necessary during pediatric airway emergencies and hemodynamic instability. It creates a low-stress, psychologically safe environment that supports the rehearsal of cognitive and procedural steps related to pediatric anesthesia, enhancing trainees' confidence in their ability to apply these skills in actual clinical settings. These games improve technical and non-technical skills and engage players, according to research [20]. Therefore, incorporating gamification into pediatric anesthesia curricula will enhance trainees' engagement, reinforce fundamental pediatric anesthesia concepts, and ultimately contribute to enhanced patient safety.

Collaborative and experiential learning methods

**Figure 3:**
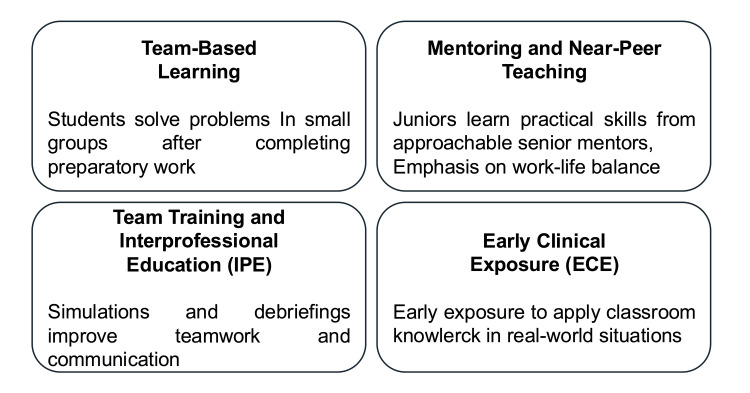
Collaborative And Experiential Learning Methods Image credits: Created by the authors using Microsoft PowerPoint (Microsoft Corp., Redmond, WA, USA).

Collaborative and experiential learning strategies have gained increasing attention in health professions education for their ability to promote active engagement, contextual reasoning, and professional identity formation. Contemporary learners often respond favorably to interactive environments that combine structured guidance with practical relevance. Within pediatric anesthesia, where crisis anticipation, teamwork, and communication are central, such approaches may be particularly valuable. The revised pediatric anesthesia curriculum may include the following methods to improve learning (Figure 3).

Team-Based Learning (TBL)

TBL is an instructional model in which small groups engage in structured problem-solving while maintaining individual accountability. Evidence from health professions education suggests that TBL enhances knowledge application, participation, communication skills, and learner satisfaction when compared with traditional lecture-based instruction [22]. Narrative syntheses also indicate improvements in collaborative reasoning and team performance, particularly when cases are thoughtfully designed, and facilitators are trained to ensure inclusive discussion. In pediatric anesthesia training, TBL may be applied through structured case analyses, for example, managing an emergency laparotomy in a child with a full stomach, requiring trainees to anticipate airway challenges, assign perioperative roles, and plan crisis responses. Such exercises reflect the multidisciplinary coordination inherent to the operating room. However, effective implementation depends on careful facilitation, as dominant participants may inadvertently limit engagement of quieter trainees without structured moderation.

Teaching and Mentoring Near-Peers

Near-peer teaching and structured mentorship represent additional experiential strategies supported by educational research. Studies in medical education report that near-peer models can enhance learner confidence, approachability of instruction, and leadership development among senior trainees [23,24]. Mentorship frameworks have also been associated with improved professional development and well-being. In pediatric anesthesia, senior fellows teaching neonatal airway skills to junior residents may create psychologically safe environments for procedural rehearsal. Formal mentorship pairings linking trainees with both faculty and senior residents can facilitate goal setting, competency progression, and reflective feedback. Reverse mentoring, wherein technologically adept trainees assist faculty with digital platforms, may further promote intergenerational collaboration. Nevertheless, the quality and consistency of near-peer initiatives depend on structured oversight and institutional support.

Team Training and Interprofessional Education (IPE)

IPE is another collaborative approach with demonstrated educational benefits. Systematic reviews indicate that IPE can improve learners’ attitudes toward teamwork and clarify professional roles, although robust long-term patient outcome data remain limited [25]. In pediatric anesthesia, where perioperative care relies on seamless coordination among anesthesiologists, surgeons, nurses, pediatricians, and respiratory therapists, interprofessional simulations and mock resuscitation drills may strengthen communication and shared situational awareness. Such initiatives may also reduce hierarchical barriers and better prepare trainees for high-acuity pediatric crises. However, logistical complexity and entrenched institutional structures may limit sustainability unless supported by organizational commitment.

Early Clinical Exposure (ECE)

ECE further integrates experiential learning into training pathways. Evidence from medical education suggests that structured early exposure enhances contextual understanding, confidence, and professional identity formation [26]. In pediatric anesthesia, supervised early participation in preoperative assessments, inductions, and family communication may help trainees link physiological principles to real-time clinical decisions. Exposure to anxious children and families may also foster empathy and communication skills. Importantly, early engagement must be carefully scaffolded to avoid cognitive overload or premature responsibility, ensuring patient safety while promoting progressive autonomy.

Ethical, psychosocial, and professional considerations

Pediatric anesthesia education also includes emotional intelligence, moral reasoning, empathetic communication, and sensitivity toward children and their families, along with the technical aspects of pediatric anesthesia. The "train without harm” philosophy, along with other core ethical principles, emphasizes the need to simulate pediatric anesthesia procedures before performing them on patients. It is equally important to create a safe and supportive environment where learners can make mistakes and learn from them, which can be fostered through well-designed debriefings, confidentiality, and non-judgmental reflection. Modern training programs should also address digital professionalism, including how to act responsibly on social media, protect patient confidentiality, and avoid inadvertently sharing digital information. Communication with pediatric patients must be age-appropriate, gentle, and engaging, using simple language, opportunities for shared decision-making, and anxiety-reducing strategies, such as the use of music or virtual reality. All these factors ensure that future pediatric anesthesiologists acquire technical, humanistic, and ethical skills.

Assessment and feedback mechanisms

Continuous assessment for Gen Z students is vital because they want feedback that is quick, helpful, and measurable. Simple, real-time corrective cues and microfeedback during clinical tasks can also help refine skills without interrupting workflow. At the same time, rapid debriefings after simulations or pediatric cases help reinforce key learning points. A fair and accurate way to evaluate skills is through competency-based assessments, such as the Objective Structured Clinical Examination (OSCE), which evaluates clinical reasoning, communication, and decision-making through standardized stations, while the Objective Structured Assessment of Technical Skills (OSATS) assesses procedural competence using structured checklists and global rating scales and Entrustable Professional Activities (EPAs) for airway management, vascular access, regional anesthesia, or neonatal resuscitation [26]. Providing faculty-guided review at regular intervals is very important for trainees, using reflection logs and digital portfolios, to document clinical exposure, simulation performance, strengths, and skill deficits. These systems promote openness, self-awareness, and purposeful skill development, all of which are very important to Gen Z’s culture of always trying to get better.

Institutional infrastructure and faculty support

Faculty development and strong institutional support are highly essential for any educational change to last. Modern, technology-enhanced training requires a robust infrastructure that includes simulation centers, child-sized mannequins, VR resources, reliable high-speed digital platforms, and dedicated technical staff. Teachers need support through workshops on designing microlearning, teaching online, and giving effective debriefings. They also need recognition systems that reward new teaching methods. Lastly, scheduling structures need to include protected time for simulation and flipped-classroom activities, as well as balanced clinical rotations that include early exposure to pediatrics and increasing independence. These program-level components ensure that an educational framework designed for the learning needs of Gen Z is consistent, scalable, and sustainable in the long run.

Barriers and challenges in pediatric anesthesia training

Gaps in Infrastructure

Some well-funded and some poorly funded academic centers have better access to pediatric simulation facilities, high-fidelity manikins, and stable internet connections. To make sure that everyone has the same chances to learn, all locations need simulation infrastructure, IT support, and maintenance of training equipment for students.

Faculty Preparedness

If senior academics don’t know how to use new teaching tools, don’t have time, or don’t trust them, they might not use them. Regular faculty development seminars, digital mentorship programs, and recognition prizes can help teachers feel more confident and excited about using e-learning, VR, and simulations.

Getting the Right Balance Between Depth and Brevity

Microlearning gets people’s attention, but too much fragmentation makes it hard to learn about complicated pediatric physiology, pharmacology, and airway issues. Including short micro-modules along with long-term case-based discussions, journal clubs, and supervised clinical reflection helps maintain a structured curriculum that maintains depth and critical thinking.

Digital Fatigue and Getting Involved

Spending too much time on screens and in virtual sessions may lead to “Zoom fatigue” or digital burnout. Short tests, reflective debriefings, peer conversations, and occasional in-person seminars are all interactive formats that can engage people and involve them.

Fairness and Inclusion

An inequality might exist among trainees who have more gadgets, more bandwidth, or are more comfortable using more advanced digital tools. Institutions should ensure that everyone can take part and find a way to learn that works for them, through hybrid learning, downloadable modules, and in-person seminars.

Gaps in the literature and future directions

Although steps are being taken to improve anesthesia education, there still isn’t enough research on how to teach Gen Z about pediatric anesthesia. Much of what we know about Gen Z’s learning patterns comes from medical or nursing school. There are still gaps in key areas, including pediatric airway management, neonatal resuscitation, congenital cardiac anesthesia, pediatric pain management, and crisis response. Promising research is emerging on digital tools such as VR, simulation, gamification, and microlearning. Still, it has not been proven in controlled pediatric settings, with limited long-term data on skill retention, safety outcomes, or real-world error reduction. Few studies directly link innovative teaching methods to improved patient outcomes, and there's no established framework that guides Gen Z learners on the optimal combination of simulation, e-learning, and bedside instruction. Ethical and psychosocial competencies, including effective communication with anxious parents, digital professionalism, and the maintenance of psychological safety in simulations, are inadequately explored. People often forget about faculty readiness and institutional barriers, especially in low-resource or low- and middle-income country settings, even though they have a big impact on how things get done. Finally, there aren’t many good long-term studies that follow Gen Z trainees through their training. To create a useful, evidence-based educational framework for the next generation of pediatric anesthesiologists, it will be necessary to address these gaps.

## Conclusions

Gen Z is a group that is changing the way people learn, which means that pediatric anesthesia training needs to change in a meaningful way. Evidence indicates that the most efficacious methodologies incorporate experiential learning, structured simulation, competency-based advancement, and supportive mentorship within psychologically secure settings. Instead of depending mostly on apprenticeship models that are focused on observation, teachers should focus on methods that encourage active engagement, clinical relevance, and ongoing formative feedback. Focusing on communication, professionalism, and reflective practice in addition to technical skills may help trainees get ready for the challenges of pediatric care. In practical terms, learner-centered training environments that combine guided clinical experience with focused educational support are most likely to produce confident, skilled, and patient-centered anesthesiologists.

## References

[1] Bhananker SM, Ramamoorthy C, Geiduschek JM (2007). Anesthesia-related cardiac arrest in children: update from the Pediatric Perioperative Cardiac Arrest Registry. Anesth Analg.

[2] Jimenez N, Posner KL, Cheney FW, Caplan RA, Lee LA, Domino KB (2007). An update on pediatric anesthesia liability: a closed claims analysis. Anesth Analg.

[3] Morray JP, Geiduschek JM, Caplan RA, Posner KL, Gild WM, Cheney FW (1993). A comparison of pediatric and adult anesthesia closed malpractice claims. Anesthesiology.

[4] Devi V, Pallath V, Gayathri B, Patil SS (2025). Perceived learning gaps in paediatric anaesthesia training: a cross-sectional survey. Indian J Anaesth.

[5] Seemiller C, Grace M, Dal Bo Campagnolo P, Mara Da Rosa Alves I, Severo De Borba G (2019). How Generation Z college students prefer to learn: a comparison of U.S. and Brazil students. Journal of Educational Research and Practice.

[6] Vizcaya-Moreno MF, Pérez-Cañaveras RM (2020). Social media used and teaching methods preferred by Generation Z students in the nursing clinical learning environment: a cross-sectional research study. Int J Environ Res Public Health.

[7] Priyanshu Priyanshu, Barik S, Dubepuria A, Mohabey A, Madegowda A, Das LS (2025). Need for generational shift in teaching methods and its application in orthopedics: why medical education should keep up with Generation Z. J Clin Orthop Trauma.

[8] Huang YT, Addab S, Bertolizio G, Hamdy R, Thorstad K, Tsimicalis A (2025). Use of virtual reality in the pediatric perioperative setting and for induction of anesthesia: mixed methods pilot feasibility study. JMIR Perioper Med.

[9] Wolbrink TA, Burns JP (2012). Teaching trainees to perform procedures on critically ill children: ethical concerns and emerging solutions. AMA J Ethics.

[10] Burnett GW, Goldhaber-Fiebert SN (2024). The role of simulation training in patients' safety in anaesthesia and perioperative medicine. BJA Educ.

[11] Daly Guris RJ, George P, Gurnaney HG (2024). Simulation in pediatric anesthesiology: current state and visions for the future. Curr Opin Anaesthesiol.

[12] Patel SM, Singh D, Hunsberger JB (2020). An advanced boot camp for pediatric anesthesiology Fellows. J Educ Perioper Med.

[13] Lorello GR, Cook DA, Johnson RL, Brydges R (2014). Simulation-based training in anaesthesiology: a systematic review and meta-analysis. Br J Anaesth.

[14] Kim KJ (2024). Medical student needs for e-learning: perspectives of the generation Z. Korean J Med Educ.

[15] Ambardekar AP, Eriksen W, Ferschl MB, McNaull PP, Cohen IT, Greeley WJ, Lockman JL (2023). A consensus-driven approach to redesigning graduate medical education: the pediatric anesthesiology Delphi study. Anesth Analg.

[16] Hew KF, Lo CK (2018). Flipped classroom improves student learning in health professions education: a meta-analysis. BMC Med Educ.

[17] Spaic D, Bukumiric Z, Rajovic N (2025). The flipped classroom in medical education: systematic review and meta-analysis. J Med Internet Res.

[18] Alsaid B, Al-Bitar A, Mousa L (2025). Short social media videos as a supplementary educational resource in neuroanatomy: a nonrandomized clinical trial. JAMA Netw Open.

[19] Cascella M, Cascella A, Monaco F, Shariff MN (2023). Envisioning gamification in anesthesia, pain management, and critical care: basic principles, integration of artificial intelligence, and simulation strategies. J Anesth Analg Crit Care.

[20] Huang WD, Loid V, Sung JS (2024). Reflecting on gamified learning in medical education: a systematic literature review grounded in the Structure of Observed Learning Outcomes (SOLO) taxonomy 2012-2022. BMC Med Educ.

[21] Joshi T, Budhathoki P, Adhikari A, Poudel A, Raut S, Shrestha DB (2022). Team-based learning among health care professionals: a systematic review. Cureus.

[22] Pierce B, van de Mortel T, Allen J, Mitchell C (2024). The influence of near-peer teaching on undergraduate health professional students' self-efficacy beliefs: a systematic integrative review. Nurse Educ Today.

[23] Haidet P, Kubitz K, McCormack WT (2014). Analysis of the team-based learning literature: TBL comes of age. J Excell Coll Teach.

[24] Spaulding EM, Marvel FA, Jacob E (2021). Interprofessional education and collaboration among healthcare students and professionals: a systematic review and call for action. J Interprof Care.

[25] Abdulrahman K (2025). Enhancing clinical education for health professions students: evidence-based strategies. Med Res Arch.

[26] Ganzhorn A, Schulte-Uentrop L, Küllmei J, Zöllner C, Moll-Khosrawi P (2023). National consensus on entrustable professional activities for competency-based training in anaesthesiology. PLoS One.

